# The effect of targeted treatment on people with patellofemoral pain: a pragmatic, randomised controlled feasibility study

**DOI:** 10.1186/s12891-017-1698-7

**Published:** 2017-08-04

**Authors:** Benjamin T. Drew, Philip G. Conaghan, Toby O. Smith, James Selfe, Anthony C. Redmond

**Affiliations:** 1 Leeds Institute of Rheumatic and Musculoskeletal Medicine, Chapel Allerton Hospital, Chapeltown Rd, Leeds, LS7 4SA UK; 2 NIHR Leeds Biomedical Research Centre, Leeds, UK; 3 School of Health Sciences, University of East Anglia, Norwich, UK; 4 Department of Health Professions, Manchester Metropolitan University, Manchester, UK

**Keywords:** Knee, Strength training, Therapeutic exercise, Patellofemoral pain, Hip strengthening

## Abstract

**Background:**

Targeted treatment, matched according to specific clinical criteria e.g. hip muscle weakness, may result in better outcomes for people with patellofemoral pain (PFP). However, to ensure the success of future trials, a number of questions on the feasibility of a targeted treatment need clarification. The aim of the study was to explore the feasibility of matched treatment (MT) compared to usual care (UC) management for a subgroup of people with PFP determined to have hip weakness and to explore the mechanism of effect for hip strengthening.

**Methods:**

In a pragmatic, randomised controlled feasibility study, 24 participants with PFP (58% female; mean age 29 years) were randomly allocated to receive either MT aimed specifically at hip strengthening, or UC over an eight-week period. The primary outcomes were feasibility outcomes, which included rates of adherence, attrition, eligibility, missing data and treatment efficacy. Secondary outcomes focused on the mechanistic outcomes of the intervention, which included hip kinematics.

**Results:**

Conversion to consent (100%), missing data (0%), attrition rate (8%) and adherence to both treatment and appointments (>90%) were deemed successful endpoints. The analysis of treatment efficacy showed that the MT group reported a greater improvement for the Global Rating of Change Scale (62% vs. 9%) and the Anterior Knee Pain Scale (−5.23 vs. 1.18) but no between-group differences for either average or worst pain. Mechanistic outcomes showed a greatest reduction in peak hip internal rotation angle for the MT group (13.1% vs. −2.7%).

**Conclusion:**

This feasibility study indicates that a definitive randomised controlled trial investigating a targeted treatment approach is achievable. Findings suggest the mechanism of effect of hip strengthening may be to influence kinematic changes in hip function in the transverse plane.

**Trial registration:**

This study was registered retrospectively. 10.1186/ISRCTN74560952. Registration date: 2017–02-06.

**Electronic supplementary material:**

The online version of this article (doi:10.1186/s12891-017-1698-7) contains supplementary material, which is available to authorized users.

## Background

Patellofemoral pain (PFP) is widely considered an enigma of musculoskeletal medicine [1]. It has been reported to affect six to 7 % of the adolescent population [2] and one in six adults who consult their GP with knee pain will be diagnosed with PFP [3]. Of major concern is that recent studies have shown that 40 to 62% [2, 4] of individuals with PFP, at one-year follow-up, report unfavourable outcomes following treatment according to current paradigms.

One solution for improving PFP therapy, proposed by the international PFP community, is to establish subgroups of the wider PFP population, to allow targeted treatment, matched according to specific subgroup characteristics [5–7]. Currently, a multimodal treatment approach (a combination of individual treatments) is recommended as best practice [8]. However, it is likely that people with PFP will benefit from being stratified and matched to specific interventions [9]. Despite successive international consensus papers since 2011 recommending subgrouping [5–7, 10], very little literature has focused on subgrouping and targeted therapy. A small number of studies [11–15] have targeted treatment based on single clinical features, although no definitive studies exist to support targeted interventions in PFP.

Reduced hip muscle strength is considered an associated feature of PFP [16]. A number of authors have reported promising clinical outcomes after prescribing hip strengthening exercises for people with PFP [17–21]. It has been proposed that individuals with PFP present with a propensity towards increased hip adduction and internal rotation during dynamic movement [22]. This is a significant predictor of pain in PFP [23], thought to be linked to increasing patellofemoral joint contact stress [24]. Subsequently, correcting this altered movement pattern is often seen as a desired outcome in interventional studies [25].

There are however, conflicting findings around the mechanistic effect of hip strengthening in PFP [25]. Some studies have demonstrated a post-interventional change in kinematics [17, 26], whilst others have reported no change [27, 28]. The reason for these conflicting findings is unclear, however, the previous studies showing no kinematic change [27, 28] have included athletic cohorts and with one of the cohorts [27] clearly showing a higher than normal baseline strength. Selfe et al. (2016) [29] recently classified people with PFP into three subgroups: ‘strong’ , ‘weak and tighter’ and ‘weak and pronated foot’. Notably, 22% were classified into the ‘strong’ subgroup with higher knee extension and hip abduction strength that may not gain from a treatment approach based on strengthening. A strengthening intervention would likely have the greatest effect on the kinematics of those with baseline weakness.

Large trials exploring a stratified approach for PFP are required, however, to ensure the success and effectiveness of such trials, a number of feasibility questions need to be answered. The primary purpose of this study was therefore to explore the feasibility of treatment matched to the specific clinical criteria of a selected subgroup compared to usual care (UC) management to inform a future stratified approach to PFP treatment. The a priori selection of a subgroup with a specific characteristic such as hip abductor weakness also provides the opportunity, as a secondary aim, to explore the mechanism of effect as this has also been recently advocated for trials of physical interventions [30].

## Methods

### Study design

The study was a pragmatic, randomised controlled feasibility study in which participants were selected from an on-going longitudinal cohort study. Twenty-six participants were identified from larger group of 70 PFP cases on the basis of having hip abductor weakness at clinical examination and were randomised into receiving either a matched treatment (MT) or UC in a 1:1 ratio. Ethical approval was obtained prior to commencement of the study (14/NE/1131). All participants completed written informed consent prior to entering the study. The study has been reported in accordance with Consolidate Standard of Reporting Trials (CONSORT) [31] and Template for Intervention Description and Replication guidelines. (TiDieR) [32].

### Participants

Participants were recruited between November 2014 to April 2016 from a large musculoskeletal and rehabilitation service via clinician referrals, their SystmOne database (a local electronic healthcare database), posters displayed in the local hospital and an university alumni volunteers website. Eligibility criteria were assessed both verbally and clinically to ensure that the inclusion criteria were addressed fully (Table 1). The most symptomatic knee was designated the index limb. Participants were stratified based on hip abductor strength measured using a Biodex isokinetic system 4 (IRPS Mediquipe, UK). Hip abductor weakness was based on thresholds defined a priori from age and gender normative data [33] (Table 1). The relevant normative mean minus one standard deviation (−1 SD) was used as the threshold for allocation to the “weak” stratum based on previous recommendations [34].

**Table 1:** Participant eligibility criteria

Inclusion criteria
• Aged 18–40 years
• Reported insidious (non-traumatic) onset of anterior or retropatellar knee pain
• Pain on two or more of the following activities: prolonged sitting, kneeling, squatting, running, patella palpation, hopping, stair walking, stepping down or isometric quadriceps contraction
• Peak hip abduction torque values [33]: Females [18–29 years] ≤ 94.1 Nm; Females [30–39 years] ≤ 75.8 Nm; Males [18–29 years] ≤ 144.1 Nm; Males [30–39 years] ≤ 139 Nm
Exclusion criteria
• Presence of inflammatory arthritis; knee pain referred from the hip or lumbar spine; any history of significant knee surgery; other causes of knee pain such as, but not restricted to: meniscal pathologies, quadriceps tendon injuries, patella tendinopathy, tibial tubercle apophysitis; bursitis
• Received any treatment within the last 3 months including physiotherapy, podiatry etc.

### Sample size

The study was designed to recruit 12 participants per group based on previous guidance for feasibility studies of this design [35]. Participants were followed up to 8 weeks post-intervention as this has previously shown to be sufficient time to demonstrate an effect in PFP [20, 27, 36].

### Randomisation

The random allocation sequence was made according to the output from a random number generator and concealed within pre-sealed, opaque envelopes [37]. All allocation and randomisation was conducted by the lead author (BD).

### Blinding

The outcome assessor was unblinded, however, patient reported outcome measures (PROMs) were completed in a separate room with no input from the assessor. The biomechanical outcomes were acquired in accordance to a strict study protocol to minimise variation and bias [38]. Furthermore, the biomechanical outputs are automated so the lack of blinding is less of an issue.

### Interventions

Participants randomised to the MT group were asked to attend six supervised sessions of approximately 30 min in duration once per week for 6 weeks at a local hospital. Each week they also performed two additional sessions on non-consecutive days independently at home, with the intervening days allowing adequate rest [39]. Consideration was made to the recommended determinants of resistance exercise [40] when developing the study intervention. The sessions were face to face, 1:1 sessions provided by the lead author (BD) a senior musculoskeletal physiotherapist with over 8 years of clinical experience. During these sessions, participants were given education and justification of the treatment followed by three exercises aimed at targeting coronal, sagittal and transverse strength of the hip using resistance bands. Each week at least one of the exercises would change with the aim of providing variation and minimising tedium [41]. The supervision sessions served as a means of ensuring both treatment fidelity and tailoring. Fidelity was ensured by checking the exercise technique and making corrections to performance prior to these being performed independently at home. Subsequent visits ensured this instruction had been correctly applied or not. Tailoring the intervention based on progressive loading was in line with current recommendations [25]. Participants were issued yellow (least resistance), red or green (most resistance) resistance tubing (66fit Ltd. ™) and were allowed to take it home. To progress the load and resistance, a Borg Rate of Perceived Exertion scale (RPE) [42] was used based on the recommendations when using resistance band [43]. A RPE of >6 was considered desirable [39] and participants were monitored after a few repetitions to ensure this was what was being achieved. As participants were stratified for strength, the intervention required participants to perform 10 repetitions within three sets as recommended for strength training [39]. Participants were advised to ensure the time under tension was 8 s (3 s concentric, 2 s isometric hold and 3 s eccentric contraction). Strengthening was performed on each leg alternatively providing a standardised rest between sets. Exercise diaries (see Additional file 1) were issued to participants to provide a reminder of the exercises and to allow a measure of adherence. Participants were asked to document each time each exercise was performed on their diary sheet and return these at each visit.

Participants randomised to the UC group continued with the same management of their condition as they were planning to receive prior to the commencement of the study. This included planned physiotherapy, podiatry or no intervention, depending upon participant preference. The type of management and number of sessions was recorded for the UC group at follow-up.

### Outcomes

In response to lack of agreed guidelines for outcomes in feasibility studies [44], the primary feasibility outcomes were adapted from recommendations made by Bugge et al. (2013) [45] and Shanyinde et al. (2011) [46].

#### Feasibility outcomes

##### Recruitment & eligibility

Recruitment and eligibility was assessed using the rate of eligibility (%), the conversion of eligible to consent (%) and a breakdown of recruitment sources.

##### Randomisation & blinding

The success of randomisation was assessed based on any problems being highlighted and whether the randomisation process yielded broad equality in both groups based on the difference in baseline characteristics. Intervention blinding is not possible for a physiotherapeutic intervention of this nature [47] and thus this could not be measured.

##### Adherence & acceptability

Adherence was assessed by the adherence rate to treatment (%) using exercise diaries and adherence to appointments (%) based on the number of ‘unable to attends’ (UTAs). The acceptability was assessed by the attrition rate (%).

##### Outcome measures

The outcome data was assessed based on the amount of missing data (%) found in each case report form.

##### Resources & study management

The study management was assessed qualitatively in terms of the logistics of running the study and the safety of all study components.

##### Treatment efficacy

PROMs provided assessments of the efficacy of the study in terms of improvements in pain and disability at eight-week follow-up. The following outcomes were selected based on current IMMPACT guidelines [48]:

1. Pain was assessed using two numerical rating scales (NRS) with a 11 point scale for: i) the average pain in the knee over the last week ii) the worst pain in the knee over the last week.
2. Function was assessed using the Anterior Knee Pain scale (AKPS), a 13-item knee specific self-reported questionnaire [49] in which 100 is the maximum achievable score and lower scores indicate greater pain and disability
3. Rating of change measured on a 11-point global rating of change scale (GROC) anchored with “very much worse” to “completely recovered” [50]. Responses were dichotomised with values greater than 0 (“unchanged”) indicating an improvement.

#### Mechanistic outcomes

The secondary aim of the study explored the potential mechanistic effects of hip strengthening on the selected sample. A selection of biomechanical variables were selected a priori to prevent subsequent data mining [51].

**Fig. 1:**
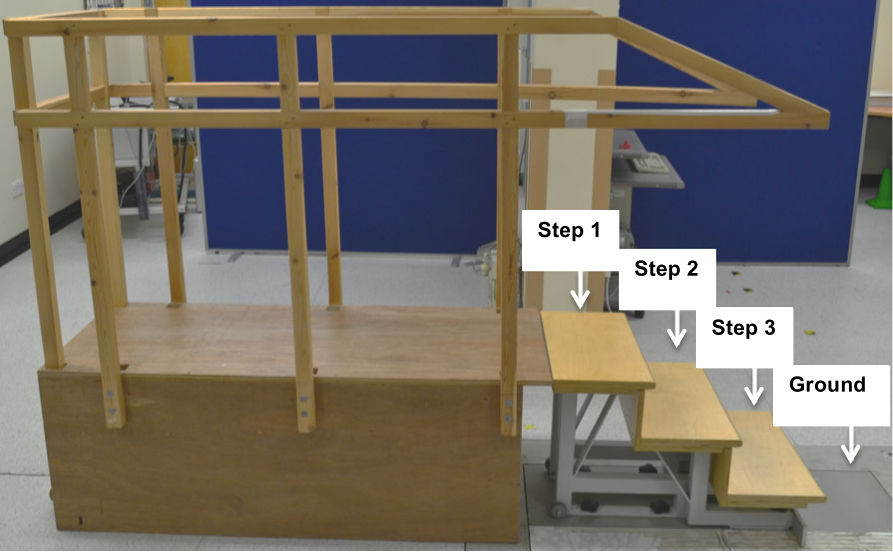
Stairs and platform used in the study. Participants descended the stairs at a self-selected speed. Each participant completed a minimum five successful stair descents. The descent was deemed successful when the index limb was placed on step two in the absence of any stumbles or hesitation. The gait cycle of interest was similar to that used in previous studies [66] between step two and ground floor. The variables of interest were captured during stance phase; between toe on and toe off on step two

Three-dimensional kinematics were assessed during stair descent using a VICON, motion capture system (Vicon Nexus Version 1.6; Vicon Motion Systems, Oxford Metrics, Oxford, Uk) at a sampling rate of 200 Hz. The stair set-up and procedure is shown Fig. 1. Stair descent was selected as this is a known aggravating factor for PFP [52], deemed challenging enough to observe a kinematic change [53] but achievable for both active and sedentary participants. Further procedural details are available in Additional file 2.

Data collected was analysed in Visual 3D (C-Motion, Rockville, Maryland). The pre-selected kinematics of most theoretical interest for explaining the proposed mechanism of action of the MT intervention were i) peak hip internal rotation angle (peak IR) of the thigh with respect to the pelvis; ii) peak hip adduction angle (peak ADD) of the thigh with respect to the pelvis; iii) total coronal hip range of movement (coronal ROM); iv) total transverse hip (ROM) (transverse ROM). These were calculated using the an X-Y-Z Cardan rotation sequence [54]. A reduction in the magnitude of all the kinematic variables measured post-intervention was considered a favourable outcome.

A Biodex isokinetic system 4 (IRPS Mediquipe, UK) was used to assess muscle strength. Both limbs were tested, with the index limb tested after a practice test using the contralateral limb. Testing the index limb second minimises any learning-effect variability [55]. To ensure accuracy, the Biodex calibration verification procedure was conducted uniformly in accordance with the system operation manual. Further procedural details are available in Additional file 3. Data was collected by Biodex Advantage Software (IRPS Mediquipe, UK). The isokinetic strength measures of interest were i) peak hip abduction torque based on the maximum hip abduction torque across five repetitions; ii) Peak knee extension torque based on the maximum knee extension torque across five repetitions.

### Statistical methods

Statistical analysis was undertaken using SPSS (version 21.0 (Armonk, NY: IBM Corp). As hypothesis testing is not advised for this size and type of study design [44], descriptive statistics along with point estimates, confidence intervals and effect sizes were presented for all PROMs and biomechanical outcomes. Within-group changes for all kinematic variables were expressed as a percentage change of the total ROM. Feasibility outcomes were described using descriptive statistics. To determine, where possible, a quantifiable measure of the feasibility outcomes, predetermined thresholds were used to indicate either success or strategies required (Table 2). Where it was not possible to use quantitative data to demonstrate success, outcomes were reported narratively.

**Table 2:** Thresholds for feasibility outcomes

Outcome	Indicator	Successful	Unsuccessful - strategies required
Recruitment & eligibility	Conversion to consent (%)	> 90	< 90
Adherence & acceptability	Adherence rate to treatment (%)	> 90	< 90
	Adherence to appointment (%)	> 90	< 90
	Attrition rate (%)	< 10	> 10
Outcome measures	Missing data (%)	<5	>5
Treatment efficacy	Average NRS	MD > 1.5 [67]	MD < 1.5
	Worst NRS	MD > 1.5 [67]	MD < 1.5
	AKPS	MD > 8 [58]	MD < 8

*MD* mean difference, *NRS* numerical rating scale

## Results

### Feasibility outcomes

**Fig. 2:**
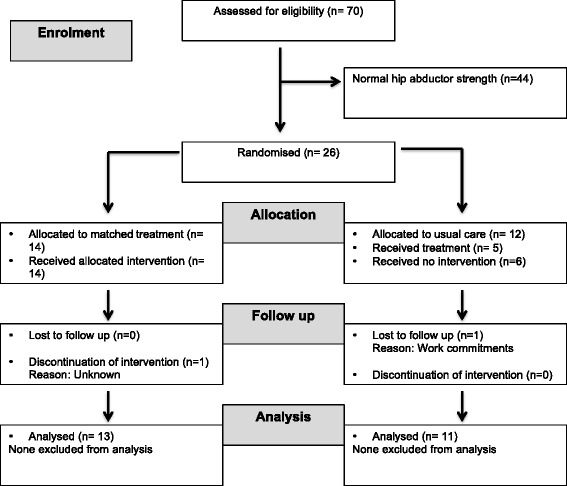
Flow of participants through the study

Figure 2 shows that 14 participants were randomised to MT and 12 participants to UC. Of the participants in the UC group, 55% received formal physiotherapy treatment, which may or may not have included a strengthening component. The remaining UC participants reported continuing with their normal self-management.

#### Recruitment & eligibility

Over 15 months, of the 70 who were screened, 26 were eligible based on hip weakness, an eligibility rate of 37.1%. All 26 eligible participants consented to the study (100% conversion to consent). Recruitment was predominantly from the SystmOne database 54% (14/26). Direct clinician referrals 15% (4/26), posters 23% (6/26) and a university alumni online advert 8% (2/26) accounted for the other sources of recruitment.

#### Randomisation and blinding

No practical problems were highlighted in the randomisation procedure. The randomisation yielded reasonable equality in terms of demographics and baseline symptoms (see Table 3). The only notable difference was the larger number of people with bilateral knee pain in the MT compared to the UC group (64% vs. 33.3% respectively).

**Table 3:** Baseline characteristics. Values are means (SD) unless stated otherwise

Characteristics	MT group (*n* = 14)	UC group (*n* = 12)
Age (years)	29.1 (6.3)	29.3 (5.5)
No (%) of females	7 (50%)	8 (66.7)
Body Mass Index (kg/m^2^)	25.9 (4.8)	27.7 (7.9)
Median (interquartile range) duration of knee pain (months)	30 (16.5–75.25)	33 (10.5–54)
Physical activity (hours/week)	3.1 (2.6)	3.9 (3.7)
No (%) with bilateral knee pain	9 (64.3)	4 (33.3)
Anterior Knee Pain Scale	74.6 (9.9)	74.75 (12.3)
Worst pain	4.7 (1.68)	5.4 (2.3)
Average pain	3.0 (1.4)	3.9 (2.2)
No of participants who had received previous treatment (%)	10 (71.4)	9 (75.0)

#### Adherence & acceptability

At post-treatment follow-up, two participants did not complete the study, an attrition rate of 8%. In the MT group, one participant did not attend their second treatment session and was then lost to contact. In the UC group, one participant was unable to complete the post-treatment analysis due to work commitments. Table 4 illustrates that of the MT group, five participants reported a 100% adherence to treatment with an overall average adherence to treatment of 94%. Treatment sessions required rearranging on seven occasions; three times for illness, three times for work commitments and once for childcare. This shows an adherence to appointment rate of 92%. Adherence to treatment and appointments was not relevant to in the UC group.

**Table 4:** Adherence to treatment for MT group

Participant	Week 1 (%)	Week 2%)	Week 3 (%)	Week 4 (%)	Week 5 (%)	Week 6 (%)	Participant adherence %
1	100	100	100	100	100	100	100
2	100	100	100	100	100	100	100
3	100	100	100	100	100	100	100
4	100	100	100	100	66.66	33.33	83.33
5	100	100	100	100	100	100	100
6	100	100	66.66	33.33	100	66.66	77.77
7	100	100	100	100	100	100	100
8	66.66	100	100	100	100	100	94.44
9	100	100	100	66.66	33.33	100	83.33
10	100	100	100	66.66	100	100	94.44
11	66.66	100	100	100	100	100	94.44
12	100	100	100	100	66.66	100	94.44
13^a^	-	-	-	-	-	-	-
14	100	100	88.88	100	100	100	98.15
Weekly adherence %	94.87	100	96.58	96.58	89.74	92.31	93.87

^a^Patient 13 did not attend after the first session

#### Outcome measures

All questionnaires were completed fully without any missing data yielding a missing data indicator of 0%.

#### Treatment efficacy

Based on the GROC, overall the MT group demonstrated a larger improvement compared to UC group (61.54% vs. 9.09% respectively). The MT group demonstrated a greater improvement in AKP score compared to UC group (Mean Difference (MD) -6.41, 95% CI: 14.23, 1.41) with a medium effect size (*d =* 0.70) (see Table 5). Both worst pain NRS (−0.41; 95% CI: -1.93, 1.12) and average pain NRS (−0.02, 95% CI: -1.01.

**Table 5:** Clinical outcomes. Mean (SD) unless otherwise stated

Outcome	Group	Baseline (SD)	Post treatment (SD)	Mean difference (baseline –post) (SD)	Confidence intervals (95%)	Mean difference (MT-Control) (95% CI)	ES (*d*) (MT- control)
AKPS	MT	75.08 (10.09)	80.31 (8.66)	−5.23 (10.17)	−11.37, 0.91	−6.41 (−14.23, 1.41)	0.70
	UC	73.64 (12.23)	72.45 (16.94)	1.18 (7.91)	−4.13, 6.49		
Worst NRS	MT	4.85 (1.68)	4.62 (2.10)	0.23 (2.05)	−1.01, 1.47	−0.41 (−1.93, 1.12)	0.23
	UC	5.27 (2.33)	4.64 (2.16)	0.64 (1.43)	−0.33, 1.59		
Average NRS	MT	3.08 (1.38)	2.46 (1.33)	0.62 (1.33)	−0.19, 1.42	−0.02 (−1.01, 0.96)	0.02
	UC	3.73 (2.19)	3.09 (1.87)	0.64 (0.92)	0.02, 2.28		
GROC	MT		61.5% (8/13)				
	UC		9.1% (1/11)				

*AKP* anterior knee pain scale, *MT* matched treatment, *UC* usual care group, *NRS* numerical rating scale, *GROC* global rating of change scale, *ES* effect size

#### Resources & Study Management

The 9% of appointments that needed rescheduling required time to make these changes. No safety issues were reported.

### Mechanistic outcomes

The results from the mechanistic outcomes are shown in Table 6. Evaluation of the peak torque measures showed that both MT and UC groups showed an increase in peak hip abductor torque from baseline to follow up but no evidence of a systematic effect between groups was observed (−0.63 Nm; 95% CI: -13.35, 12.09). In terms of peak knee extensor torque, the UC group showed a much larger increase yielding a MD of 7.96 Nm (95% CI -2.88, 18.79; *d* = 0.624).

**Table 6:** Mechanistic outcomes. Mean (SD) unless otherwise stated

Outcome	Group	Baseline (SD)	Post Rx (SD)	Mean difference (baseline-post) (SD)	Confidence intervals (95%)	Mean difference (MT-UC) (95% CI)	ES (*d*) (MT - UC)
Hip abductor strength (Nm)	MT	91.02 (28.45)	99.40 (27.89)	8.39 (15.28)	−17.62, 0.85	−0.63 (−13.35, 12.09)	−0.04
	UC	81.82 (31.76)	89.57 (33.43)	7.76 (14.59)	−17.56, 2.05		
Knee extensor strength (Nm)	MT	91.44 (28.21)	93.12 (27.19)	1.677 (14.57)	−10.48, 7.12	7.96 (−2.88,18.79)	0.62
	UC	94.32 (44.10)	103.95 (46.09)	9.64 (10.15)	−16.46, − 2.82		
Peak Hip Adduction (°)	MT	5.74 (2.70)	5.92 (2.79)	−0.17 (2.84)	−1.89, 1.54	−0.14 (−3.12, 2.85)	0.04
	UC	3.70 (3.68)	3.74 (4.99)	−0.04 (4.18)	−2.84, 2.77		
Peak Hip Internal Rotation (°)	MT	−4.49 (3.26)	−5.95 (5.26)	1.45 (4.98)	−1.56, 4.46	1.70 (−2.56, 5.97)	−0.34
	UC	−6.11 (4.82)	−5.86 (7.22)	−0.25 (5.06)	−3.65, 3.15		
Total coronal hip ROM (°)	MT	9.77 (3.62)	9.29 (2.60)	0.47 (2.19)	−0.86, 1.79	1.12 (−0.72, 3.06)	−0.53
	UC	10.04 (4.69)	10.74 (4.79)	−0.70 (2.27)	−2.23, 0.82		
Total transverse hip ROM (°)	MT	11.08 (2.65)	11.39 (2.08)	−0.32 (2.49)	−1.83, 1.19	0.46 (−1.45, 2.38)	−0.20
	UC	9.12 (5.76)	9.90 (5.02)	−0.78 (1.93)	−2.08, 0.52		

*MT* matched treatment, *UC* usual care group, *ROM* range of movement, *ES* effect size

The between-group comparisons of the kinematics showed that the MT group had a reduction in peak IR whereas the UC had a slight increase (1.70°; 95% CI: −2.56, 5.97) yielding a small effect size (*d* = −0.34). Both MT and UC groups showed an increase in peak ADD (−0.17° vs. -0.04° respectively). Coronal ROM showed that the MT group had a reduction whereas the UC group showed a slight increase (1.12°; 95% CI: −0.72, 3.06) yielding a medium effect size (*d* = −0.53). Transverse ROM showed an increase in both the MT and UC groups (−0.32° vs. -0.78° respectively.

**Fig. 3:**
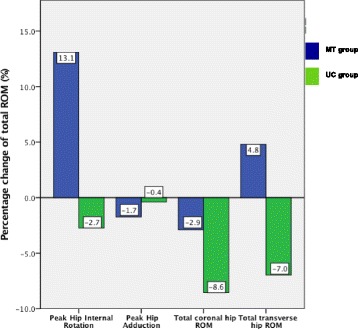
Percentage change of total ROM in kinematic outcomes between baseline and post intervention

The within-group comparisons of the kinematic outcomes are presented in Fig. 3. The MT intervention led to a reduction in peak IR of 13.1% of the total transverse ROM. There was a small reduction in coronal ROM (4.8%) whilst peak ADD and transverse ROM demonstrated a small increase. The UC group demonstrated an increase for all kinematic variables.

## Discussion

The aims of this study were: i) to determine the feasibility of treatment matched to the specific characteristics of selected PFP sub group and ii) to explore the proposed mechanism of effect of employing strengthening in a subgroup with baseline hip weakness. A definitive randomised controlled trial (RCT) appears achievable in terms of adherence, attrition, eligibility and outcome data. Some consideration is required to develop strategies to enhance the ability to quantify clinical differences between groups. In terms of the potential mechanism of effect for hip strengthening, an improvement was shown for peak IR following MT.

### Feasibility outcomes

Based on our eligibility thresholds for selecting a ‘weak’ hip group, we predetermined that for feasibility; eligibility should reach or exceed 32%. Our observed eligibility rate of 37% provides reassurance. It is also expected that other people with PFP, ineligible for the current study based on hip strength, may well classify into other subgroups as shown in recent studies [29]. This eligibility rate for hip weakness is less than the 88% (at 1SD) reported by Selfe et al. (2016) [29] but may be explained by the current study measuring an isokinetic contraction rather than an isometric contraction and as a consequence the different strength thresholds applied. Furthermore in order to minimise potential bias, future multicentre RCTs would need to ensure cross-site calibration of the isokinetic systems and site visits to monitor fidelity of the testing procedures and the intervention.

Recent studies have reported that a greater adherence to treatment is associated with increased probability of better outcomes [2]. An adherence to treatment and adherence to appointments over 90% is promising. Approximately 30% (4/13) achieved complete adherence to all treatment sessions and 9% of appointments required rearranging. Our adherence rate is comparable to a larger RCT [18] who found a 80.3% adherence rate for a 6 week hip strengthening in PFP. It’s anticipated that rearranging appointments for participants would be more challenging for a larger sample over multiple sites. Consequently, strategies to enhance adherence with the use of activity monitoring technology and reminder services need to be considered [56].

No differences between groups were found for either the average or worst NRS values. This might be explained by the difference in almost a score of one in average baseline NRS, a feature that would likely be minimised in a larger full-scale trial. Previous RCTs [18, 57] have also used eligibility criteria requiring a minimum NRS score of three out of 10 pain score. Setting a minimum pain score as part of the inclusion criteria is suggested for a future RCT.

The difference between groups for the AKP score did not reach the predetermined minimal clinically important difference (MCID) of eight points [58], although there was a trend towards a meaningful benefit. These findings are similar to the only other RCT to have stratified a PFP cohort, used in a study of a foot orthotic intervention over 6 weeks. Mills et al. (2011) [12] selected their participants based on predictors shown to predict success with orthotics, which included age, height, baseline pain severity and a static foot measure. They also found a significant difference between groups in terms of GROC with no differences in AKP score or VAS pain. They suggest that GROC is able to capture the multidimensional nature of PFP (characterised by pain, disability and functional limitation) compared to AKP score and VAS pain which are more one dimensional [12].

### Mechanistic outcomes

Stratifying for hip abductor weakness led to a reduction of approximately 13% for peak IR in the MT group following strengthening treatment. This is important considering that an increase peak IR has been associated with PFP during stair descent [59, 60]. This reduction in peak IR occurred with a slight worsening of their transverse ROM suggesting that following treatment, people in the MT group were initiating stance phase in a more desirable externally rotated hip position. A reduction in peak IR and a slight increase in peak ADD are perhaps surprising considering that participants were stratified for hip abductor weakness. However, recent strength measures conducted on 501 healthy athletes [61] have shown that hip abductor and hip external rotation strength are highly correlated (*r* = 0.66) indicating this subgroup were likely to have also demonstrated weakness into both hip abduction and external rotation.

Hip strength increased in both groups by a similar difference, which is likely the result of over a third of participants in the UC group being engaged in physiotherapy. Post-hoc analysis of those participants in the UC group who received *no treatment* show an increase of only 3.9 Nm in hip abductor strength (results not shown). In the MT group the change in hip strength was 9%, which is a comparable improvement to previous hip strengthening programmes over a similar training duration [21, 27]. The increase in strength for both groups but with only kinematic improvements seen in the MT group might suggest that strength, on its own, cannot explain the improvement seen in peak IR. Direct comparison with previous studies [17, 26–28] remains difficult due to the differences in assessment tasks (e.g. running, stairs etc.) and the specific kinematic outcomes (e.g. peak, average angles etc.) investigated. Previous studies [27, 28] that have observed the effect of hip strengthening on running kinematics in people with PFP found no change in kinematics despite increases in hip abductor strength. Only Baldon et al. (2014) [17] reported changes in kinematics, during a single leg squat, following a hip strengthening programme. Yet, this training programme, did include constant feedback on lower limb alignment which suggests a more movement retraining approach [62] rather than pure strength training. It remains possible that the improvements observed in peak IR in the current study was the result of using progressive loading within a tailored treatment regime and selecting participants who were most likely to benefit from strengthening.

### Limitations

This feasibility study presents with several limitations. Firstly, the study was performed in a single centre. Future RCTs would be required to be multicentre to improve generalisability, which is anticipated to introduce new feasibility issues. The findings of the current study would, however, inform the documentation of standard operating procedures in terms of recruitment; data collection and intervention provision to ensure any future study could be operationalised across different geographical locations. Secondly, the current study did not blind assessors to group allocation, which could lead to potential bias [63]. Every effort was made for participants to complete PROMs in isolation and objective biomechanical outcomes were acquired in accordance with strict protocols with little chance of introducing bias. Future RCTs should make every effort to introduce outcome assessor blinding and consider measuring the level of this outcome assessor blinding [64] Thirdly, the use of a UC group was intended to represent the heterogeneity of management available in real, daily practice and thus improve the external validity [65]. For the purposes of exploring the mechanism of hip strengthening, however, the fact that over half of the UC group received physiotherapy input potentially dilutes the between-group findings. Comparison with a control group receiving no active intervention would remedy this issue.

## Conclusion

The potential benefits associated with stratification and subgrouping within PFP have been advocated since the first International Patellofemoral Pain Retreat Consensus statement [10]. This study suggests that targeted treatment provides a greater improvement in overall function and self-reported improvement in comparison to usual care. Additionally, the improvements seen in peak IR following MT suggest this may be a plausible mechanism of effect for hip strengthening when treatment is matched to an appropriate subgroup. Strategies to enhance the ability to detect clinical difference should be considered and might be improved by selection of participants with a minimum pain score. Ultimately, a pragmatic, multicentre RCT with a sufficiently powered cohort appears achievable and should be conducted to determine the clinical and cost-effectiveness of a stratified treatment approach versus usual care for people with PFP.

## Additional files

Additional file 1: Home Exercise Training Diary. (PDF 1211 kb)

Additional file 2: Procedures for capturing 3D kinematics. (PDF 72 kb)

Additional file 3: Procedures for obtaining strength. (PDF 74 kb)

## Abbreviations

3D: three dimensional
AKPS: anterior knee pain scale
CONSORT: consolidated standards of reporting trials
Coronal ROM: total coronal hip range of movement
GP: general practitioner
GROC: global rating of change scale
MCID: minimal
MT: matched treatment
Nm: Newton metre
NRS: numerical rating scale
Peak ADD: peak hip adduction angle
Peak IR: peak hip internal rotation angle
PFP: patellofemoral pain
PROM: patient reported outcome measures
RCT: randomised controlled trial
ROM: range of movement
RPE: rate of perceived exertion
SD: standard deviation
TiDieR: template for intervention description and replication guidelines
Transverse ROM: total transverse hip range of movement
UC: usual care
UTA: unable to attend IMMPACT Initiative on Methods, Measurements and Pain Assessment in Clinical Trials
