# Supplementary material for: The effect of targeted treatment on people with patellofemoral pain: a pragmatic, randomised controlled feasibility study
Source: BMC Musculoskelet Disord. 2017 Aug 4;18:338. doi: 10.1186/s12891-017-1698-7 (PMC5545020; doi:10.1186/s12891-017-1698-7)
Supplement: Supplementary file 1 — Home Exercise Training Diary. (PDF 1211 kb) [file 12891_2017_1698_MOESM1_ESM.pdf]

UNIVERSITY OF LEEDS

The Leeds Teaching Hospitals NHS
NHS Trust

NHS
National Institute for Health Research

WEEK ONE – Home Exercise Training Diary

Please perform the marked exercises three times per week (including the supervised session). Mark when the exercises are completed – there are seven boxes, one for each day of the week.
Rest for 2 minutes between sets

To complete	Exercise	Description	Week	Sets	Repetitions	To mark when completed	% Completed
	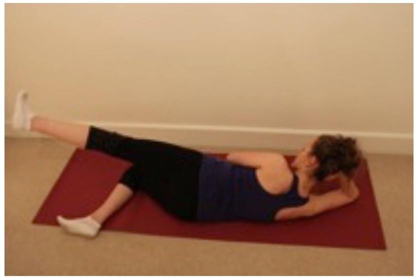	1. Lying on your side with painful leg on top<br>2. Ensure your leg is in a neutral position<br>3. Slowly lift your leg upwards	Week 1				
			Week 2				
			Week 3				
			Week 4				
			Week 5				
			Week 6				
	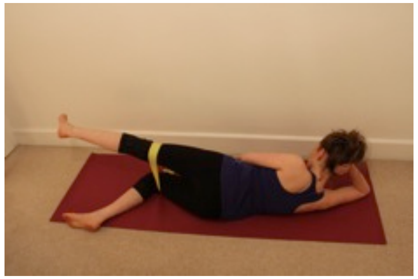	1. Lying on your side with painful leg on top<br>2. Ensure your leg is in a neutral position<br>3. Tie yellow band around both ankles<br>4. Slowly lift your leg upwards against band	Week 1				
			Week 2				
			Week 3				
			Week 4				
			Week 5				
			Week 6				

Patient number | | | |

Date of issue | D | D | M | M | Y | Y |

WEEK ONE – Home Exercise Training Diary

Please perform the <u>marked</u> exercises three times per week (including the supervised session). Mark when the exercises are completed – there are seven boxes, one for each day of the week.
Rest for 2 minutes between sets

	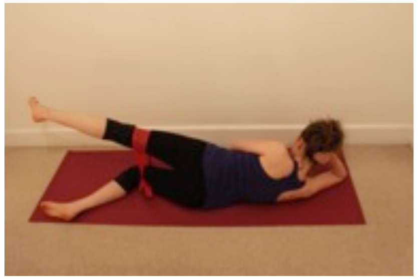	1. Lying on your side with painful leg on top<br>2. Ensure your leg is in a neutral position<br>3. Tie red band around both ankles<br>4. Slowly lift your leg upwards against band	Week 1										
			Week 2										
			Week 3										
			Week 4										
			Week 5										
			Week 6										
	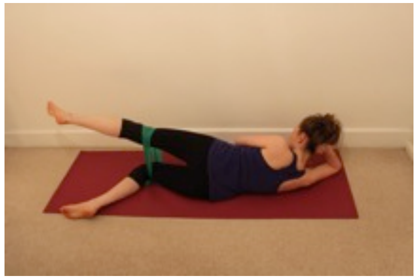	1. Lying on your side with painful leg on top<br>2. Ensure your leg is in a neutral position<br>3. Tie green band around both ankles<br>4. Slowly lift your leg upwards against band	Week 1										
			Week 2										
			Week 3										
			Week 4										
			Week 5										
			Week 6										

Patient number | | | |

Date of issue | D | D | M | M | Y | Y |

University of Leeds | The Leeds Teaching Hospitals NHS Trust | NHS National Institute for Health Research

Week One – Home Exercise Training Diary

Please perform the marked exercises three times per week (including the supervised session). Mark when the exercises are completed – there are seven boxes, one for each day of the week.

Rest for 2 minutes between sets

To complete	Exercise	Description	Week	Sets	Repetitions	To mark when completed	% Completed
	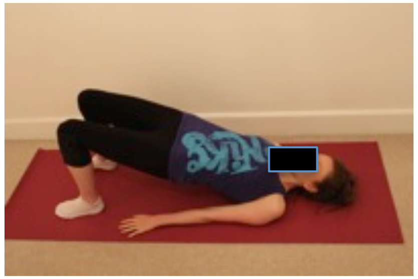	1. Lying on your back with knees bent<br>2. Slowly lift your buttocks off the floor<br>3. Then slowly lower to the floor	Week 1				
			Week 2				
			Week 3				
			Week 4				
			Week 5				
			Week 6				
	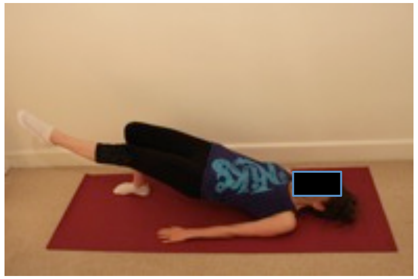	1. Lying on your back with knees bent<br>2. Bend the non-painful leg to the chest<br>3. Then with painful leg on the floor, squeeze the buttocks and lift buttocks off the floor<br>4. Then slowly lower to the floor	Week 1				
			Week 2				
			Week 3				
			Week 4				
			Week 5				
			Week 6				

Patient number

Date of issue D D M M Y Y

WEEK ONE – Home Exercise Training Diary

Please perform the marked exercises three times per week (including the supervised session). Mark when the exercises are completed – there are seven boxes, one for each day of the week.
Rest for 2 minutes between sets

	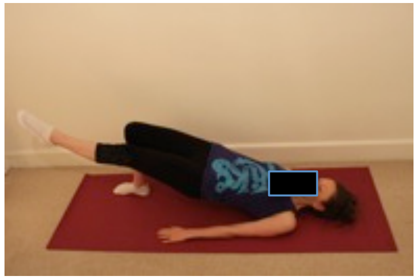	1. Lying on your back with knees bent<br>2. Bend the non-painful leg to the chest<br>3. Then with painful leg on the floor, squeeze the buttocks and lift buttocks off the floor<br>4. Then slowly lower to the floor<br>5. Hold for 10 seconds	Week 1									
			Week 2									
			Week 3									
			Week 4									
			Week 5									
			Week 6									

Patient number | | | |

Date of issue | D | D | M | M | Y | Y |

WEEK TWO – Home Exercise Training Diary

Please perform the marked exercises three times per week (including the supervised session). Mark when the exercises are completed – there are seven boxes, one for each day of the week.

Rest for 2 minutes between sets

To complete	Exercise	Description	Week	Sets	Repetitions	To mark when completed	% Completed
	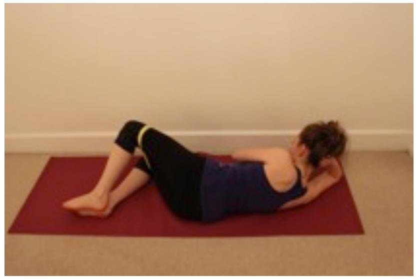	1. Lying on your side with painful leg on top<br>2. Keep ankles and knees together with your thighs at 45 to your body<br>3. Tie yellow band around the lower thighs<br>4. Keeping ankles together slowly lift top upwards<br>5. Keep your pelvis still and ensure you don't rotate the rest of your body<br>Return to beginning	Week 1				
			Week 2				
			Week 3				
			Week 4				
			Week 5				
			Week 6				
	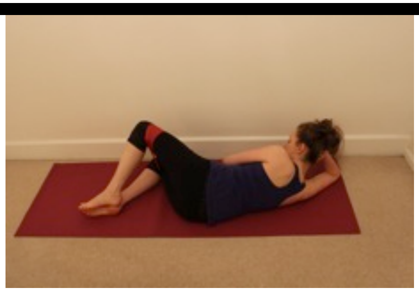	1. Lying on your side with painful leg on top<br>2. Keep ankles and knees together with your thighs at 45 to your body<br>3. Tie red band around the lower thighs<br>4. Keeping ankles together slowly lift top leg upwards<br>5. Keep your pelvis still and ensure	Week 1				
			Week 2				
			Week 3				
			Week 4				
			Week 5				

Patient Number [ ][ ][ ]

Date of issue D D M M Y Y

WEEK TWO – Home Exercise Training Diary

Please perform the marked exercises three times per week (including the supervised session). Mark when the exercises are completed – there are seven boxes, one for each day of the week.
Rest for 2 minutes between sets

		you don't rotate the rest of your body<br>Return to beginning	Week 6										
	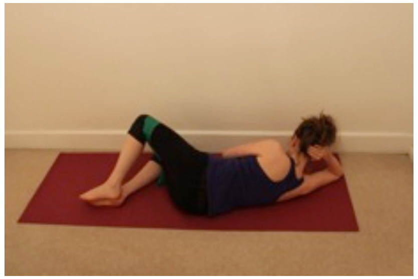	1. Lying on your side with painful leg on top<br>2. Keep ankles and knees together with your thighs at 45 to your body<br>3. Tie green band around the lower thighs<br>4. Keeping ankles together slowly lift top leg upwards<br>5. Keep your pelvis still and ensure you don't rotate the rest of your body<br>6. Return to beginning	Week 1										
			Week 2										
			Week 3										
			Week 4										
			Week 5										
			Week 6										

UNIVERSITY OF LEEDS

The Leeds Teaching Hospitals NHS
NHS Trust

NHS National Institute for Health Research

WEEK TWO – Home Exercise Training Diary

Please perform the marked exercises three times per week (including the supervised session). Mark when the exercises are completed – there are seven boxes, one for each day of the week.

Rest for 2 minutes between sets

To complete	Exercise	Description	Week	Sets	Repetitions	To mark when completed	% Completed
	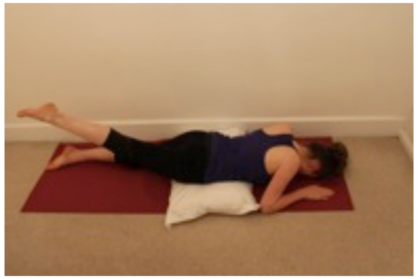	1. Lying on your front over a pillow positioned at waist level<br>2. Keep your leg straight and lift upwards from the hip then return<br>3. Try not to rotate your body	Week 1				
			Week 2				
			Week 3				
			Week 4				
			Week 5				
			Week 6				
	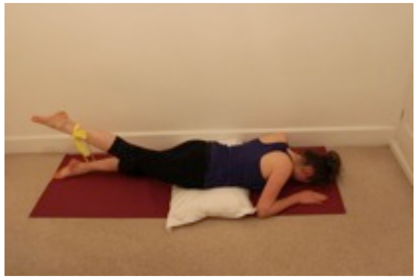	1. Lying on your front over a pillow positioned at waist level<br>2. Tie yellow band around ankle<br>3. Keep your leg straight and lift upwards from the hip then return<br>4. Try not to rotate your body	Week 1				
			Week 2				
			Week 3				
			Week 4				
			Week 5				
			Week 6				

Patient Number

Date of issue D D M M Y Y

WEEK TWO – Home Exercise Training Diary

Please perform the <u>marked</u> exercises three times per week (including the supervised session). Mark when the exercises are completed – there are seven boxes, one for each day of the week.
Rest for 2 minutes between sets

	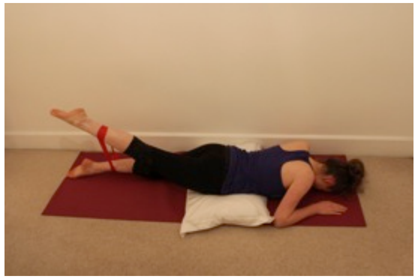	1. Lying on your front over a pillow positioned at waist level<br>2. Tie red band around ankle<br>3. Keep your leg straight and lift upwards from the hip then return<br>4. Try not to rotate your body	Week 1										
			Week 2										
			Week 3										
			Week 4										
			Week 5										
			Week 6										
	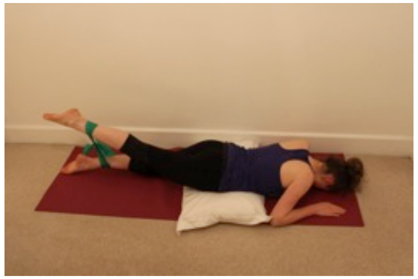	1. Lying on your front over a pillow positioned at waist level<br>2. Tie green band around ankle<br>3. Keep your leg straight and lift upwards from the hip then return<br>4. Try not to rotate your body	Week 1										
			Week 2										
			Week 3										
			Week 4										
			Week 5										
			Week 6										

Patient Number | | | |

Date of issue | D | D | M | M | Y | Y |

UNIVERSITY OF LEEDS

The Leeds Teaching Hospitals NHS Trust

National Institute for Health Research

WEEK TWO – Home Exercise Training Diary

Please perform the marked exercises three times per week (including the supervised session). Mark when the exercises are completed – there are seven boxes, one for each day of the week.

Rest for 2 minutes between sets

To complete	Exercise	Description	Week	Sets	Repetitions	To mark when completed	% Completed
	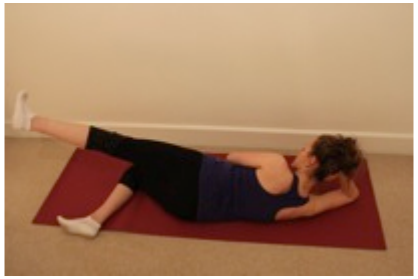	1. Lying on your side with painful leg on top<br>2. Ensure your leg in neutral position<br>3. Slowly lift your leg upwards	Week 1				
			Week 2				
			Week 3				
			Week 4				
			Week 5				
			Week 6				
	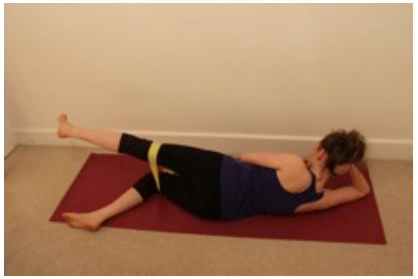	1. Lying on your side with painful leg on top<br>2. Ensure your leg in neutral position<br>3. Tie yellow band around both ankles<br>4. Slowly lift your leg upwards against band	Week 1				
			Week 2				
			Week 3				
			Week 4				
			Week 5				
			Week 6				

Patient Number

Date of issue D D M M Y Y

WEEK TWO – Home Exercise Training Diary

Please perform the marked exercises three times per week (including the supervised session). Mark when the exercises are completed – there are seven boxes, one for each day of the week.
Rest for 2 minutes between sets

	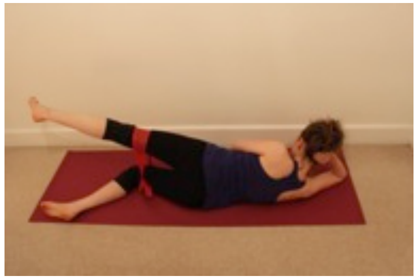	1. Lying on your side with painful leg on top<br>2. Ensure your leg in neutral position<br>3. Tie red band around both ankles<br>4. Slowly lift your leg upwards against band	Week 1										
			Week 2										
			Week 3										
			Week 4										
			Week 5										
			Week 6										
	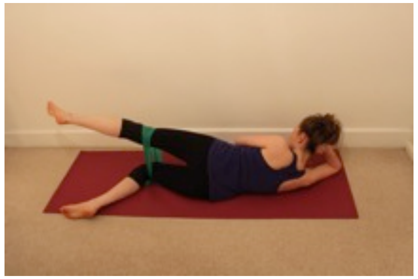	1. Lying on your side with painful leg on top<br>2. Ensure your leg in neutral position<br>3. Tie green band around both ankle<br>4. Slowly lift your leg upwards against band	Week 1										
			Week 2										
			Week 3										
			Week 4										
			Week 5										
			Week 6										

Patient Number | | |

Date of issue | D | D | M | M | Y | Y |

WEEK TWO – Home Exercise Training Diary

Please perform the <u>marked</u> exercises three times per week (including the supervised session). Mark when the exercises are completed – there are seven boxes, one for each day of the week.
Rest for 2 minutes between sets

To complete	Exercise	Description	Week	Sets	Repetitions	To mark when completed	% Completed
	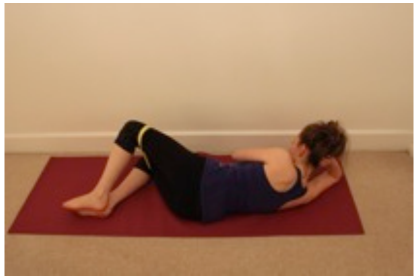	1. Lying on your side with painful leg on top<br>2. Keep ankles and knees together with your thighs at 45 to your body<br>3. Tie yellow band around the lower thighs<br>4. Keeping ankles together slowly lift top upwards<br>5. Keep your pelvis still and ensure you don't rotate the rest of your body<br>6. Return to beginning	Week 1				
			Week 2				
			Week 3				
			Week 4				
			Week 5				
			Week 6				
	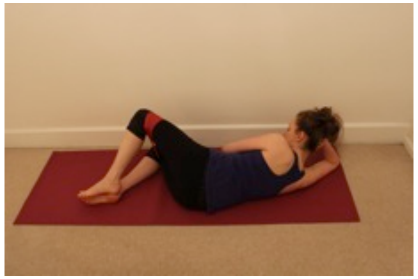	1. Lying on your side with painful leg on top<br>2. Keep ankles and knees together with your thighs at 45 to your body<br>3. Tie red band around the lower thighs<br>4. Keeping ankles together slowly lift top leg upwards<br>5. Keep your pelvis still and ensure you don't rotate the rest of your body	Week 1				
			Week 2				
			Week 3				
			Week 4				
			Week 5				
			Week 6				

Patient Number | | | |

Date of issue D D M M Y Y

WEEK TWO – Home Exercise Training Diary

Please perform the marked exercises three times per week (including the supervised session). Mark when the exercises are completed – there are seven boxes, one for each day of the week.
Rest for 2 minutes between sets

		6. Return to beginning											
	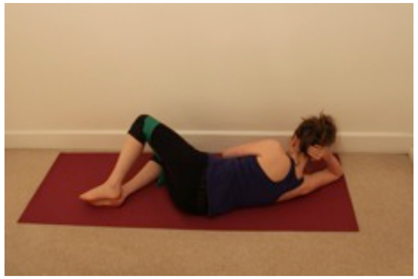	1. Lying on your side with painful leg on top<br>2. Keep ankles and knees together with your thighs at 45 to your body<br>3. Tie green band around the lower thighs<br>4. Keeping ankles together slowly lift top leg upwards<br>5. Keep your pelvis still and ensure you don't rotate the rest of your body<br>6. Return to beginning	Week 1										
			Week 2										
			Week 3										
			Week 4										
			Week 5										
			Week 6										

Patient Number | | |

Date of issue | D | D | M | M | Y | Y |

UNIVERSITY OF LEEDS

The Leeds Teaching Hospitals NHS Trust

NHS National Institute for Health Research

Week Three – Home Exercise Training Diary

Please perform the <u>marked</u> exercises three times per week (including the supervised session). Mark when the exercises are completed – there are seven boxes, one for each day of the week.
Rest for 2 minutes between sets

To complete	Exercise	Description	Week	Sets	Repetitions	To mark when completed							% Completed
	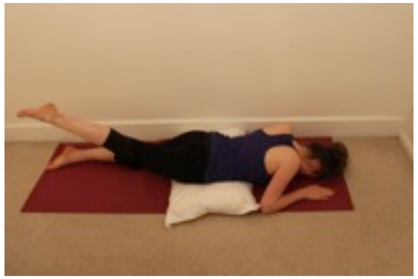	1. Lying on your front over a pillow positioned at waist level<br>2. Keep your leg straight and lift upwards from the hip then return<br>3. Try not to rotate your body	Week 1										
			Week 2										
			Week 3										
			Week 4										
			Week 5										
			Week 6										
	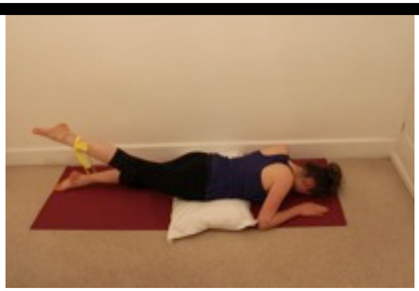	1. Lying on your front over a pillow positioned at waist level<br>2. Tie yellow band around ankle<br>3. Keep your leg straight and lift upwards from the hip then return<br>4. Try not to rotate your body	Week 1										
			Week 2										
			Week 3										
			Week 4										
			Week 5										

Patient Number | | | |

Date of issue | D | D | M | M | Y | Y |

UNIVERSITY OF LEEDS

The Leeds Teaching Hospitals NHS
NHS Trust

NHS
National Institute for
Health Research

WEEK THREE – Home Exercise Training Diary

Please perform the marked exercises three times per week (including the supervised session). Mark when the exercises are completed – there are seven boxes, one for each day of the week.
Rest for 2 minutes between sets

			Week 6										
	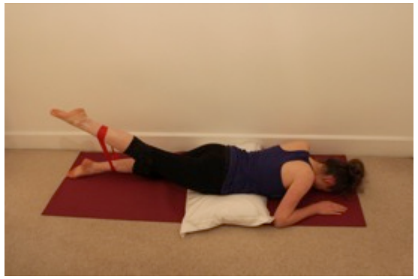	1. Lying on your front over a pillow positioned at waist level<br>2. Tie red band around ankle<br>3. Keep your leg straight and lift upwards from the hip then return<br>4. Try not to rotate your body	Week 1										
			Week 2										
			Week 3										
			Week 4										
			Week 5										
			Week 6										
	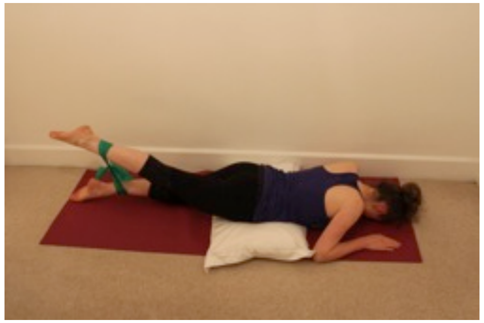	1. Lying on your front over a pillow positioned at waist level<br>2. Tie green band around ankle<br>3. Keep your leg straight and lift upwards from the hip then return<br>4. Try not to rotate your body	Week 1										
			Week 2										
			Week 3										
			Week 4										
			Week 5										
			Week 6										

Patient Number | | | |

Date of issue | D | D | M | M | Y | Y |

WEEK THREE – Home Exercise Training Diary

Please perform the marked exercises three times per week (including the supervised session). Mark when the exercises are completed – there are seven boxes, one for each day of the week.
Rest for 2 minutes between sets

To complete	Exercise	Description	Week	Sets	Repetitions	To mark when completed							% Completed
	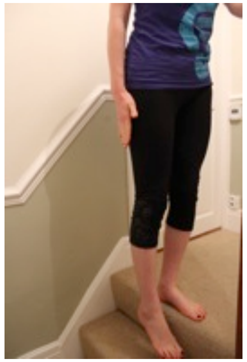	1. Standing on the lateral step on your painful leg (use support if necessary)<br>2. With the non-painful leg let this drop towards the floor<br>4. Tighten buttocks on painful leg side, lifting your non-painful leg back to neutral	Week 1										
			Week 2										
			Week 3										
			Week 4										
			Week 5										
			Week 6										
	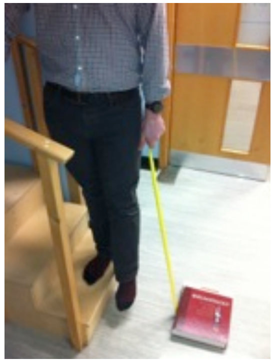	1. Standing on the lateral step on your painful leg (use support if necessary)<br>2. Secure the yellow theraband to the floor with a weight e.g. heavy book<br>3. With the non-painful leg let this drop towards the floor<br>4. Tighten buttocks on painful leg side, lifting your non-painful leg back to neutral	Week 1										
			Week 2										
			Week 3										
			Week 4										
			Week 5										
			Week 6										

Patient Number | | | |

Date of issue | D | D | M | M | Y | Y |

WEEK THREE – Home Exercise Training Diary

Please perform the marked exercises three times per week (including the supervised session). Mark when the exercises are completed – there are seven boxes, one for each day of the week.
Rest for 2 minutes between sets

	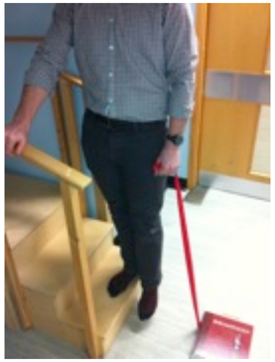	1. Standing on the lateral step on your painful leg (use support if necessary)<br>2. Secure the red theraband to the floor with a weight e.g. heavy book<br>3. With the non-painful leg let this drop towards the floor<br>4. Tighten buttocks on painful leg side, lifting your non-painful leg back to neutral	Week 1											
			Week 2											
			Week 3											
			Week 4											
			Week 5											
			Week 6											
	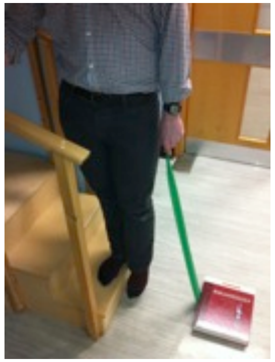	1. Standing on the lateral step on your painful leg (use support if necessary)<br>2. Secure the green theraband to the floor with a weight e.g. heavy book<br>3. With the non-painful leg let this drop towards the floor<br>4. Tighten buttocks on painful leg side, lifting your non-painful leg back to neutral	Week 1											
			Week 2											
			Week 3											
			Week 4											
			Week 5											
			Week 6											

Patient Number | | | |

Date of issue | D | D | M | M | Y | Y |

UNIVERSITY OF LEEDS

The Leeds Teaching Hospitals NHS Trust

NHS National Institute for Health Research

WEEK THREE – Home Exercise Training Diary

Please perform the marked exercises three times per week (including the supervised session). Mark when the exercises are completed – there are seven boxes, one for each day of the week.

Rest for 2 minutes between sets

To complete	Exercise	Description	Week	Sets	Repetitions	To mark when completed	% Completed
	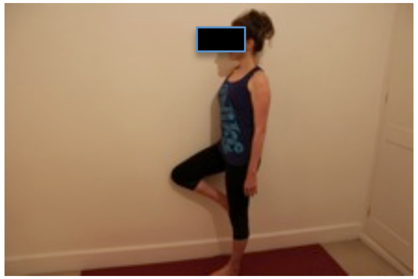	1. Standing on your painful leg with your non-painful leg against a wall<br>2. Bend your non-painful knee up and push into the wall<br>3. Keep the leg and pelvis in neutral<br>4. Push and then relax	Week 1				
			Week 2				
			Week 3				
			Week 4				
			Week 5				
			Week 6				
	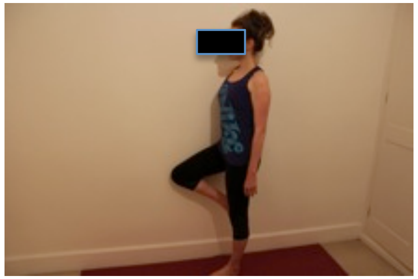	1. Standing on your painful leg with your non-painful leg against a wall<br>2. Bend your non-painful knee up and push into the wall<br>3. Keep the leg and pelvis in neutral<br>4. 4. Hold 15 seconds	Week 1				
			Week 2				
			Week 3				
			Week 4				
			Week 5				

Patient Number | | |

Date of issue D D M M Y Y

Week Three – Home Exercise Training Diary

Please perform the marked exercises three times per week (including the supervised session). Mark when the exercises are completed – there are seven boxes, one for each day of the week.

Rest for 2 minutes between sets

			Week 6										
	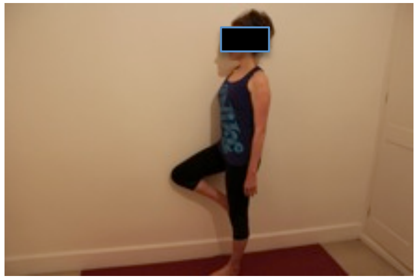	1. Standing on your painful leg with your non-painful leg against a wall<br>2. Bend your non-painful knee up and push into the wall<br>3. Keep the leg and pelvis in neutral<br>4. Hold 30 seconds	Week 1										
			Week 2										
			Week 3										
			Week 4										
			Week 5										
			Week 6										

Patient Number | | | |

Date of issue | D | D | M | M | Y | Y |

University of Leeds | The Leeds Teaching Hospitals NHS Trust | NHS National Institute for Health Research

WEEK FOUR– Home Exercise Training Diary

Please perform the marked exercises three times per week (including the supervised session). Mark when the exercises are completed – there are seven boxes, one for each day of the week.
Rest for 2 minutes between sets

To complete	Exercise	Description	Week	Sets	Repetitions	To mark when completed	% Completed
	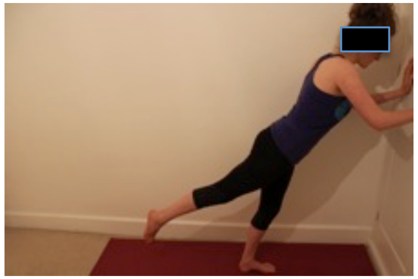	1. Standing upright supported with a slight forward lean<br>2. Slowly take the painful leg backwards at the hip<br>3. Try not rotate the body	Week 1				
			Week 2				
			Week 3				
			Week 4				
			Week 5				
			Week 6				
	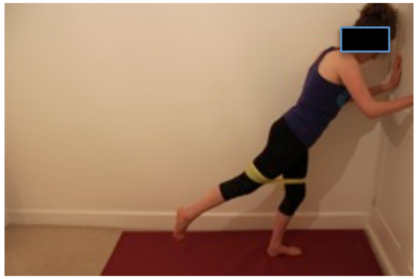	1. Standing upright supported with a slight forward lean<br>2. Tie a yellow band around the ankles<br>3. Slowly take the painful leg backwards at the hip<br>4. Try not rotate the body	Week 1				
			Week 2				
			Week 3				
			Week 4				
			Week 5				
			Week 6				

Patient Number

Date of issue D D M M Y Y

UNIVERSITY OF LEEDS

The Leeds Teaching Hospitals NHS
NHS Trust

NHS National Institute for Health Research

WEEK FOUR– Home Exercise Training Diary

Please perform the marked exercises three times per week (including the supervised session). Mark when the exercises are completed – there are seven boxes, one for each day of the week.
Rest for 2 minutes between sets

	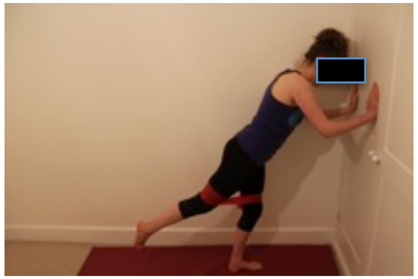	1. Standing upright supported with a slight forward lean<br>2. Tie a red band around the ankles<br>3. Slowly take the painful leg backwards at the hip<br>4. Try not rotate the body	Week 1										
			Week 2										
			Week 3										
			Week 4										
			Week 5										
			Week 6										
	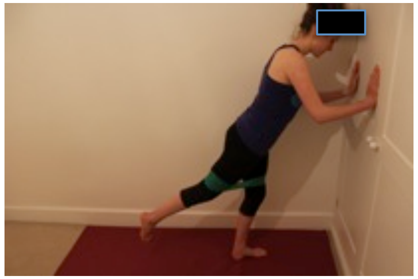	1. Standing upright supported with a slight forward lean<br>2. Tie a red band around the ankles<br>3. Slowly take the painful leg backwards at the hip<br>4. Try not rotate the body	Week 1										
			Week 2										
			Week 3										
			Week 4										
			Week 5										
			Week 6										

Patient Number | | | |

Date of issue | D | D | M | M | Y | Y |

UNIVERSITY OF LEEDS

The Leeds Teaching Hospitals NHS
NHS Trust

NHS
National Institute for Health Research

WEEK FOUR– Home Exercise Training Diary

Please perform the marked exercises three times per week (including the supervised session). Mark when the exercises are completed – there are seven boxes, one for each day of the week.

Rest for 2 minutes between sets

To complete	Exercise	Description	Week	Sets	Repetitions	To mark when completed	% Completed
	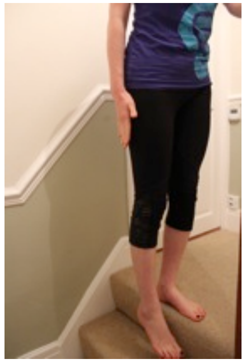	1. Standing on the lateral step on your painful leg (use support if necessary)<br>2. With the non-painful leg let this drop towards the floor<br>3. Tighten buttocks on painful leg side, lifting your non-painful leg back to neutral	Week 1				
			Week 2				
			Week 3				
			Week 4				
			Week 5				
			Week 6				
	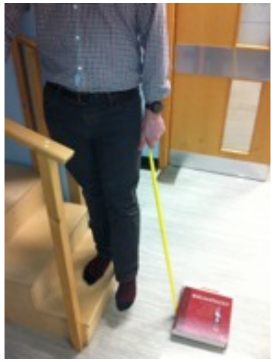	1. Standing on the lateral step on your painful leg (use support if necessary)<br>2. Secure the yellow theraband to the floor with a weight e.g. heavy book<br>3. With the non-painful leg let this drop towards the floor<br>4. Tighten buttocks on painful leg side, lifting your non-painful leg back to neutral	Week 1				
			Week 2				
			Week 3				
			Week 4				
			Week 5				
			Week 6				

Patient Number

Date of issue D D M M Y Y

UNIVERSITY OF LEEDS

The Leeds Teaching Hospitals NHS Trust

NHS National Institute for Health Research

WEEK FOUR– Home Exercise Training Diary

Please perform the marked exercises three times per week (including the supervised session). Mark when the exercises are completed – there are seven boxes, one for each day of the week.
Rest for 2 minutes between sets

	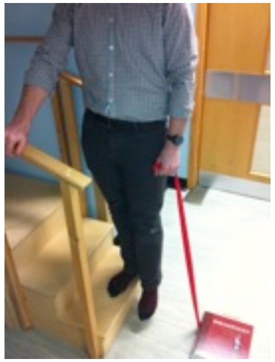	1. Standing on the lateral step on your painful leg (use support if necessary)<br>2. Secure the red theraband to the floor with a weight e.g. heavy book<br>3. With the non-painful leg let this drop towards the floor<br>4. Tighten buttocks on painful leg side, lifting your non-painful leg back to neutral	Week 1										
			Week 2										
			Week 3										
			Week 4										
			Week 5										
			Week 6										
	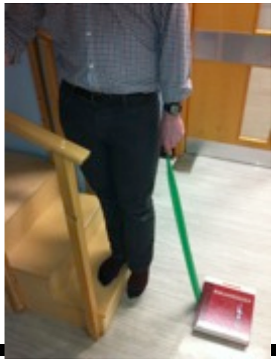	1. Standing on the lateral step on your painful leg (use support if necessary)<br>2. Secure the green theraband to the floor with a weight e.g. heavy book<br>3. With the non-painful leg let this drop towards the floor<br>4. Tighten buttocks on painful leg side, lifting your non-painful leg back to neutral	Week 2										
			Week 3										
			Week 4										
			Week 5										
			Week 6										

Patient Number

Date of issue D D M M Y Y

WEEK FIVE – Home Exercise Training Diary

Please perform the marked exercises three times per week (including the supervised session). Mark when the exercises are completed – there are seven boxes, one for each day of the week.

Rest for 2 minutes between sets

To complete	Exercise	Description	Week	Sets	Repetitions	To mark when completed							% Completed
	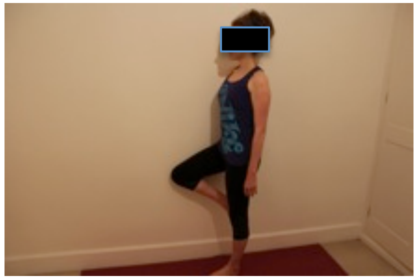	1. Standing on your painful leg with your non-painful leg against a wall<br>2. Bend your non-painful knee up and push into the wall<br>3. Keep the leg and pelvis in neutral<br>4. Push and then relax	Week 1										
			Week 2										
			Week 3										
			Week 4										
			Week 5										
			Week 6										
	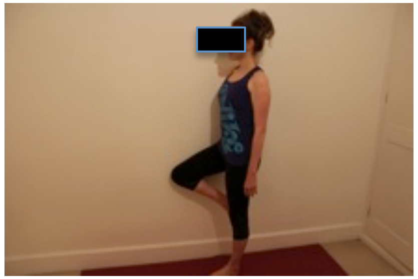	1. Standing on your painful leg with your non-painful leg against a wall<br>2. Bend your non-painful knee up and push into the wall<br>3. Keep the leg and pelvis in neutral<br>4. 4. Hold 15 seconds	Week 1										
			Week 2										
			Week 3										
			Week 4										
			Week 5										
			Week 6										

Patient Number | | |

Date of issue | D | D | M | M | Y | Y |

UNIVERSITY OF LEEDS

The Leeds Teaching Hospitals NHS
NHS Trust

NHS
National Institute for Health Research

WEEK FIVE – Home Exercise Training Diary

Please perform the marked exercises three times per week (including the supervised session). Mark when the exercises are completed – there are seven boxes, one for each day of the week.
Rest for 2 minutes between sets

	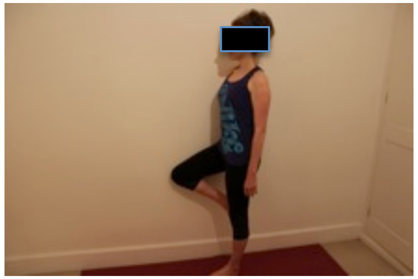	1. Standing on your painful leg with your non-painful leg against a wall<br>2. Bend your non-painful knee up and push into the wall<br>3. Keep the leg and pelvis in neutral<br>4. Hold 30 seconds	Week 1											
			Week 2											
			Week 3											
			Week 4											
			Week 5											
			Week 6											

Patient Number | | | |

Date of issue | D | D | M | M | Y | Y |

UNIVERSITY OF LEEDS

The Leeds Teaching Hospitals NHS Trust

National Institute for Health Research

Week Five – Home Exercise Training Diary

Please perform the <u>marked</u> exercises three times per week (including the supervised session). Mark when the exercises are completed – there are seven boxes, one for each day of the week.
Rest for 2 minutes between sets

To complete	Exercise	Description	Week	Sets	Repetitions	To mark when completed	% Completed
	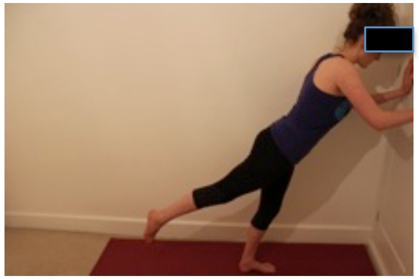	1. Standing upright supported with a slight forward lean<br>2. Slowly take the painful leg backwards at the hip<br>3. Try not rotate the body	Week 1				
			Week 2				
			Week 3				
			Week 4				
			Week 5				
			Week 6				
	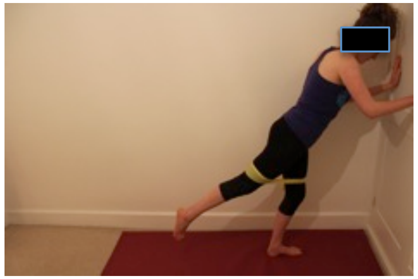	1. Standing upright supported with a slight forward lean<br>2. Tie a yellow band around the ankles<br>3. Slowly take the painful leg backwards at the hip<br>4. Try not rotate the body	Week 1				
			Week 2				
			Week 3				
			Week 4				
			Week 5				
			Week 6				

Patient Number | | | |

Date of issue D D M M Y Y

UNIVERSITY OF LEEDS

The Leeds Teaching Hospitals NHS Trust

NHS National Institute for Health Research

WEEK FIVE – Home Exercise Training Diary

Please perform the <u>marked</u> exercises three times per week (including the supervised session). Mark when the exercises are completed – there are seven boxes, one for each day of the week.
Rest for 2 minutes between sets

	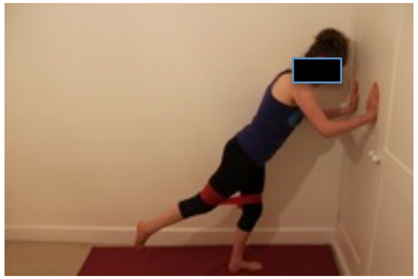	1. Standing upright supported with a slight forward lean<br>2. Tie a red band around the ankles<br>3. Slowly take the painful leg backwards at the hip<br>4. Try not rotate the body	Week 1										
			Week 2										
			Week 3										
			Week 4										
			Week 5										
			Week 6										

	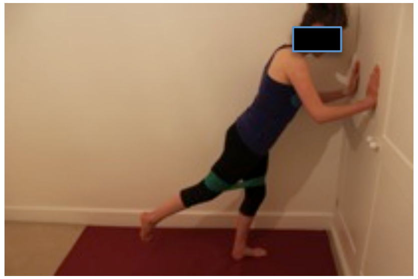	1. Standing upright supported with a slight forward lean<br>2. Tie a red band around the ankles<br>3. Slowly take the painful leg backwards at the hip<br>4. Try not rotate the body	Week 1										
			Week 2										
			Week 3										
			Week 4										
			Week 5										
			Week 6										

Patient Number | | | |

Date of issue | D | D | M | M | Y | Y |

UNIVERSITY OF LEEDS

The Leeds Teaching Hospitals NHS Trust

National Institute for Health Research

Week Five – Home Exercise Training Diary

Please perform the marked exercises three times per week (including the supervised session). Mark when the exercises are completed – there are seven boxes, one for each day of the week.

Rest for 2 minutes between sets

To complete	Exercise	Description	Week	Sets	Repetitions	To mark when completed							% Completed
	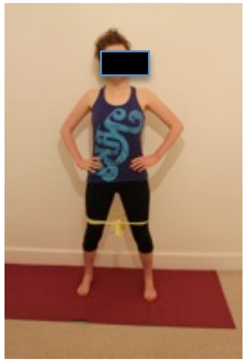	1. Standing with feet shoulder width apart<br>2. Tie yellow band around ankles<br>3. Maintain a 30 degree hip and knee position<br>4. Side step one direction then the other<br>5. Keep tension on the band and toes pointing forwards	Week 1										
			Week 2										
			Week 3										
			Week 4										
			Week 5										
			Week 6										
	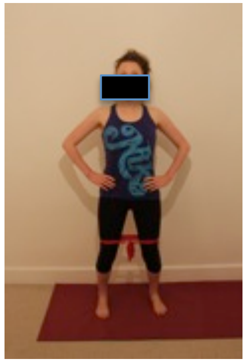	1. Standing with feet shoulder width apart<br>2. Tie red band around ankles<br>3. Maintain a 30 degree hip and knee position<br>4. Side step one direction then the other<br>5. Keep tension on the band and toes pointing forwards	Week 1										
			Week 2										
			Week 3										
			Week 4										
			Week 5										
			Week 6										

Patient Number | | | |

Date of issue | D | D | M | M | Y | Y |

UNIVERSITY OF LEEDS

The Leeds Teaching Hospitals NHS Trust

NHS National Institute for Health Research

WEEK FIVE – Home Exercise Training Diary

Please perform the marked exercises three times per week (including the supervised session). Mark when the exercises are completed – there are seven boxes, one for each day of the week.

Rest for 2 minutes between sets

	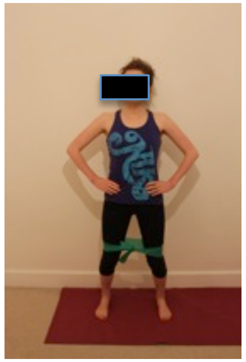	1. Standing with feet shoulder width apart<br>2. Tie green band around ankles<br>3. Maintain a 30 degree hip and knee position<br>4. Side step one direction then the other<br>5. Keep tension on the band and toes pointing forwards	Week 1										
			Week 2										
			Week 3										
			Week 4										
			Week 5										
			Week 6										

Patient Number | | | |

Date of issue | D | D | M | M | Y | Y |

UNIVERSITY OF LEEDS | The Leeds Teaching Hospitals NHS Trust | NHS National Institute for Health Research

WEEK SIX – Home Exercise Training Diary

Please perform the marked exercises three times per week (including the supervised session). Mark when the exercises are completed – there are seven boxes, one for each day of the week.
Rest for 2 minutes between sets

To complete	Exercise	Description	Week	Sets	Repetitions	To mark when completed	% Completed
	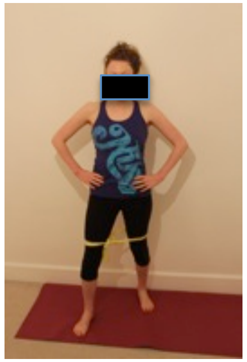	1. Standing with feet shoulder width apart<br>2. Tie yellow band around ankles<br>3. Maintain a 30 degree hip and knee position<br>4. Take diagonal, forward steps Forwards and then backwards	Week 1				
			Week 2				
			Week 3				
			Week 4				
			Week 5				
			Week 6				
	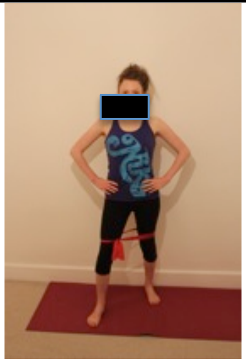	1. Standing with feet shoulder width apart<br>2. Tie red band around ankles<br>3. Maintain a 30 degree hip and knee position<br>4. Take diagonal, forward steps Forwards and then backwards	Week 1				
			Week 2				
			Week 3				
			Week 4				
			Week 5				

Patient Number

Date of issue D D M M Y Y

UNIVERSITY OF LEEDS

The Leeds Teaching Hospitals NHS Trust

NHS National Institute for Health Research

WEEK SIX – Home Exercise Training Diary

Please perform the marked exercises three times per week (including the supervised session). Mark when the exercises are completed – there are seven boxes, one for each day of the week.

Rest for 2 minutes between sets

			Week 6										
	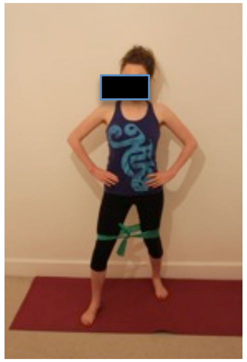	1. Standing with feet shoulder width apart<br>2. Tie green band around ankles<br>3. Maintain a 30 degree hip and knee position<br>4. Take diagonal, forward steps Forwards and then backwards	Week 1										
			Week 2										
			Week 3										
			Week 4										
			Week 5										
			Week 6										

Patient Number | | | |

Date of issue | D | D | M | M | Y | Y |

UNIVERSITY OF LEEDS

The Leeds Teaching Hospitals NHS Trust

NHS National Institute for Health Research

WEEK SIX – Home Exercise Training Diary

Please perform the marked exercises three times per week (including the supervised session). Mark when the exercises are completed – there are seven boxes, one for each day of the week.

Rest for 2 minutes between sets

To complete	Exercise	Description	Week	Sets	Repetitions	To mark when completed	% Completed
	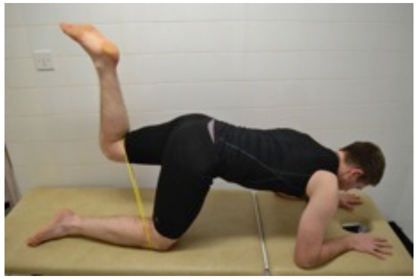	1. In the quadrupled position with the upper body supported by the forearms<br>2. Place yellow theraband around the thigh near the knee joint<br>3. Lift the leg up towards the ceiling with the knee bent to 90 degrees pulling the band	Week 1				
			Week 2				
			Week 3				
			Week 4				
			Week 5				
			Week 6				
	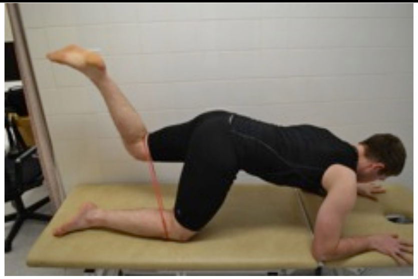	1. In the quadrupled position with the upper body supported by the forearms<br>2. Place red theraband around the thigh near the knee joint<br>3. Lift the leg up towards the ceiling with the knee bent to 90 degrees pulling the band	Week 1				
			Week 2				
			Week 3				
			Week 4				
			Week 5				

Patient Number

Date of issue D D M M Y Y

UNIVERSITY OF LEEDS

The Leeds Teaching Hospitals NHS Trust

NHS National Institute for Health Research

<u>WEEK SIX – Home Exercise Training Diary</u>

Please perform the <u>marked</u> exercises three times per week (including the supervised session). Mark when the exercises are completed – there are seven boxes, one for each day of the week.

Rest for 2 minutes between sets

			Week 6										
	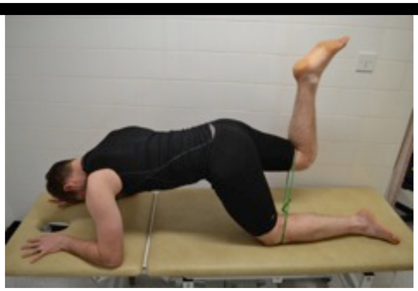	1. In the quadrupled position with the upper body supported by the forearms<br>2. Place green theraband around the thigh near the knee joint<br>3. Lift the leg up towards the ceiling with the knee bent to 90 degrees pulling the band	Week 1										
			Week 2										
			Week 3										
			Week 4										
			Week 5										
			Week 6										

Patient Number | | | |

Date of issue | D | D | M | M | Y | Y |

Week Six – Home Exercise Training Diary

Please perform the marked exercises three times per week (including the supervised session). Mark when the exercises are completed – there are seven boxes, one for each day of the week.
Rest for 2 minutes between sets

To complete	Exercise	Description	Week	Sets	Repetitions	To mark when completed	% Completed
	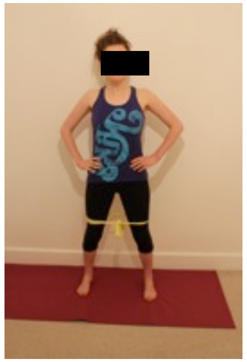	1. Standing with feet shoulder width apart<br>2. Tie yellow band around ankles<br>3. Maintain a 30 degree hip and knee position<br>4. Side step one direction then the other<br>5. Keep tension on the band and toes pointing forwards	Week 1				
			Week 2				
			Week 3				
			Week 4				
			Week 5				
			Week 6				
	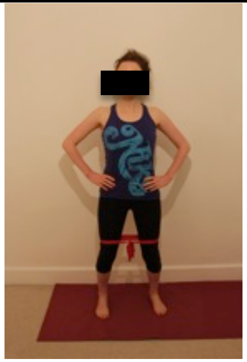	1. Standing with feet shoulder width apart<br>2. Tie red band around ankles<br>3. Maintain a 30 degree hip and knee position<br>4. Side step one direction then the other<br>5. Keep tension on the band and toes pointing forwards	Week 1				
			Week 2				
			Week 3				
			Week 4				
			Week 5				

Patient Number | | | |

Date of issue | D | D | M | M | Y | Y |

UNIVERSITY OF LEEDS

The Leeds Teaching Hospitals NHS
NHS Trust

NHS
National Institute for Health Research

WEEK SIX – Home Exercise Training Diary

Please perform the marked exercises three times per week (including the supervised session). Mark when the exercises are completed – there are seven boxes, one for each day of the week.
Rest for 2 minutes between sets

			Week 6										
	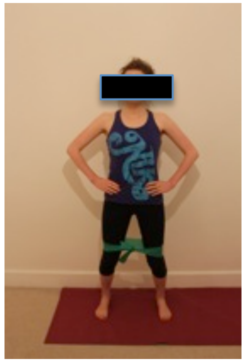	1. Standing with feet shoulder width apart<br>2. Tie green band around ankles<br>3. Maintain a 30 degree hip and knee position<br>4. Side step one direction then the other<br>5. Keep tension on the band and toes pointing forwards	Week 1										
			Week 2										
			Week 3										
			Week 4										
			Week 5										
			Week 6										

Patient Number | | | |

Date of issue | D | D | M | M | Y | Y |

UNIVERSITY OF LEEDS | The Leeds Teaching Hospitals NHS Trust | NHS National Institute for Health Research

WEEK SIX – Home Exercise Training Diary

Please perform the marked exercises three times per week (including the supervised session). Mark when the exercises are completed – there are seven boxes, one for each day of the week.
Rest for 2 minutes between sets

To complete	Exercise	Description	Week	Sets	Repetitions	To mark when completed	% Completed
	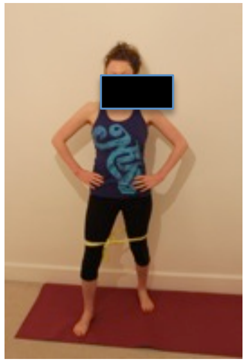	1. Standing with feet shoulder width apart<br>2. Tie yellow band around ankles<br>3. Maintain a 30 degree hip and knee position<br>4. Take diagonal, forward steps<br>5. Forwards and then backwards	Week 1				
			Week 2				
			Week 3				
			Week 4				
			Week 5				
			Week 6				
	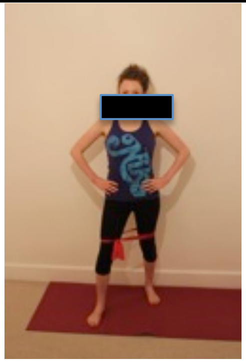	1. Standing with feet shoulder width apart<br>2. Tie red band around ankles<br>3. Maintain a 30 degree hip and knee position<br>4. Take diagonal, forward steps<br>5. Forwards and then backwards	Week 1				
			Week 2				
			Week 3				
			Week 4				
			Week 5				

Patient Number | | | |

Date of issue | D | D | M | M | Y | Y |

UNIVERSITY OF LEEDS

The Leeds Teaching Hospitals NHS
NHS Trust

NHS
National Institute for Health Research

WEEK SIX – Home Exercise Training Diary

Please perform the marked exercises three times per week (including the supervised session). Mark when the exercises are completed – there are seven boxes, one for each day of the week.
Rest for 2 minutes between sets

			Week 6										
	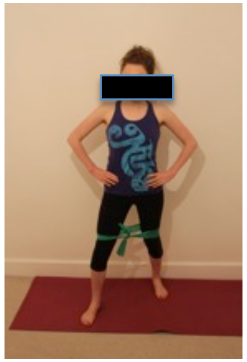	1. Standing with feet shoulder width apart<br>2. Tie green band around ankles<br>3. Maintain a 30 degree hip and knee position<br>4. Take diagonal, forward steps<br>5. Forwards and then backwards	Week 1										
			Week 2										
			Week 3										
			Week 4										
			Week 5										
			Week 6										

Patient Number | | | |

Date of issue | D | D | M | M | Y | Y |
