# Supplementary material for: The effect of targeted treatment on people with patellofemoral pain: a pragmatic, randomised controlled feasibility study
Source: BMC Musculoskelet Disord. 2017 Aug 4;18:338. doi: 10.1186/s12891-017-1698-7 (PMC5545020; doi:10.1186/s12891-017-1698-7)
Supplement: Supplementary file 2 — Procedures for capturing 3D kinematics. (PDF 72 kb) [file 12891_2017_1698_MOESM2_ESM.pdf]

Additional file 2: Procedures for capturing 3D kinematics

The marker configuration for the study was based on the calibrated anatomical systems technique (CAST) [1]. Tracking clusters, comprising of four 9mm retroreflective markers, were positioned on the lateral thighs, lateral shanks and sacrum. A static calibration trial was collected prior initiating the stairs. In order to determine anatomical reference points, retroreflective markers were attached to distal 1st metatarsal, distal 5th metatarsal, lateral and medial femoral epicondyles, right and left greater trochanters, right and left iliac crests and anterior superior iliac spines (ASIS) and posterior superior iliac spines (PSIS). Knee and ankle joint centres were calculated based on midpoint between the femoral epicondyles and malleoli respectively [2]. Hip joint centres were based on the recommendations made by Bell *et al* (1989) [3]. Additional markers were used to control for marker occlusion by the stairs which included proximal 1st metatarsal, sustenaculum tali, lateral talar process, proximal calcaneus, calcaneal tuberosity

1. Cappozzo A, Cappello A, Croce UD, and Pensalfini F. *Surface-marker cluster design criteria for 3-D bone movement reconstruction.* IEEE Trans Biomed Eng. 1997; 44(12). 1165-74.
2. Chapman GJ, Parkes MJ, Forsythe L, Felson DT, and Jones R. *Ankle motion influences the external knee adduction moment and may predict who will respond to lateral wedge insoles?: an ancillary analysis from the SILK trial.* Osteoarthritis Cartilage. 2015; 23(8). 1316-22.
3. Bell AL, Brand RA, and Pedersen DR. *Prediction of hip joint centre location from external landmarks.* Human Movement Science. 1989; 8(1). 3-16.
