# Supplementary material for: The effect of targeted treatment on people with patellofemoral pain: a pragmatic, randomised controlled feasibility study
Source: BMC Musculoskelet Disord. 2017 Aug 4;18:338. doi: 10.1186/s12891-017-1698-7 (PMC5545020; doi:10.1186/s12891-017-1698-7)
Supplement: Supplementary file 3 — Procedures for obtaining strength. (PDF 74 kb) [file 12891_2017_1698_MOESM3_ESM.pdf]

Additional file 3: Procedures for obtaining strength

For hip abduction strength testing, the dynamometer axis was arranged to centre on the hip joint. Participants were positioned in a side-lying position at a neutral angle (0° flexion, abduction, rotation) [1]. The testing protocol included three sub-maximal practice movements, similar to previous hip abduction strength testing protocols used in PFP [2]. Participants were then asked to perform five maximal effort movements from 0-30° hip abduction at a speed of 30°/s in accordance with the normative data [3]. The participant was verbally encouraged to push away and towards the pad during testing [4]. The effect of gravity on torque data was adjusted for by weighing the limb prior to each trial allowing automatic calculation. For knee extensor strength testing, participants were positioned in sitting with the knee at 90° flexion and the dynamometer axis arranged to the centre of the knee joint. The angle of testing was between 0-90° at a speed of 60°/s. The same testing regime was used as for hip abduction.

1. Souza RB and Powers CM. *Differences in hip kinematics, muscle strength, and muscle activation between subjects with and without patellofemoral pain.* J Orthop Sports Phys Ther. 2009; 39(1). 12-19.
2. Boling M and Padua D. *RELATIONSHIP BETWEEN HIP STRENGTH AND TRUNK, HIP, AND KNEE KINEMATICS DURING A JUMP ‐ LANDING TASK IN INDIVIDUALS WITH PATELLOFEMORAL PAIN.* Int J Sports Phys Ther. 2013; 8(5). 661.
3. Danneskiold ‐ Samsøe B, Bartels E, Bülow P, et al. *Isokinetic and isometric muscle strength in a healthy population with special reference to age and gender.* Acta Physiol (Oxf). 2009; 197(s673). 1-68.
4. Marchant DC, Greig M, and Scott C. *Attentional focusing instructions influence force production and muscular activity during isokinetic elbow flexions.* J Strength Cond Res. 2009; 23(8). 2358-66.
